# Measurement Repeatability and Concordance of Corneal Tomography in Keratoconus Using Anterion Versus Pentacam

**DOI:** 10.3390/bioengineering13101130

**Published:** 2026-09-28

**Authors:** Ramadhan Ahmed, Kamini Narendra Reddy, Mahad Mohamed, Nakul S. Shekhawat

**Affiliations:** 1 School of Medicine, Johns Hopkins University School of Medicine, Baltimore, MD 21205, USA; 2 Wilmer Eye Institute, Johns Hopkins University School of Medicine, Baltimore, MD 21287, USA

**Keywords:** keratoconus, anterior segment optical coherence tomography, Scheimpflug tomography, ectasia, cornea

## Abstract

**Purpose:** This study aimed to compare the repeatability and agreement of corneal tomographic measurements between the Anterion Cornea App and Pentacam HR in keratoconus (KCN). **Methods:** Eyes with KCN were imaged three consecutive times with each device. Intra-device repeatability was assessed using intraclass correlation coefficients (ICCs), within-subject standard deviation (Sw), coefficient of variation (CV), and repeatability coefficients (RCs). Inter-device agreement was evaluated using concordance correlation coefficients (CCCs) and Bland–Altman analysis. Parameters included anterior and posterior keratometry values (K1, K2, K_mean_, K_max_, and ΔK), central corneal thickness (CCT), and thinnest pachymetry (TP). **Results:** Twenty-eight eyes from 17 patients were included. Both devices demonstrated high same-session repeatability (ICC > 0.90 and CV < 5%) for all parameters except anterior ΔK and posterior ΔK. Anterion demonstrated lower measurement variability than Pentacam for all parameters. For anterior K_max_, Anterion had an RC of 1.38 diopters (D) versus 2.48 D for Pentacam. Pachymetric RCs were lower with Anterion than with Pentacam (CCT: 6.24 vs. 18.85 μm; TP: 5.93 vs. 20.34 μm). Inter-device agreement was substantial (CCC > 0.95) for most parameters except ΔK. Compared with Pentacam, Anterion measured CCT as 6.16 μm thinner and TP as 11.99 μm thinner. Bland–Altman analysis showed greater inter-device variability for all anterior keratometry parameters, posterior K1, and posterior K_mean_. **Conclusions:** Anterion demonstrated lower same-session measurement variability than Pentacam HR, particularly for K_max_ and pachymetry. Future longitudinal studies across multiple sessions are needed to assess whether Anterion’s lower same-session variability confers any advantages in KCN detection and progression monitoring. Overall, the two devices should not be used interchangeably for KCN assessment.

## 1. Introduction

Keratoconus (KCN) affects more than 23.7 million people worldwide [1]. Early corneal biometric screening can detect disease progression, identify eyes eligible for corneal collagen cross-linking (CXL), and prevent vision loss. Scheimpflug tomography is widely used for imaging eyes with KCN. Pentacam HR (Oculus, Wetzlar, Germany) measures several parameters, such as K1, K2, ΔK, K_mean_, K_max_, and thinnest pachymetry, several of which are used by cornea specialists to determine keratoconus severity and changes over time.

A key challenge in detecting KCN progression is increased measurement variability, particularly in eyes with more advanced disease. Using Pentacam HR, Kreps et al. reported reduced repeatability in 61.1% of anterior and 50% of posterior corneal parameters in eyes with mild KCN [2]. The K_max_ repeatability limit was 1.66 diopters (D) in eyes with moderate KCN, compared with 0.44 D in eyes with subclinical KCN [2]. Wadhwa et al. demonstrated greater measurement variability for K_max_ (6.25 D vs. 1.69 D) and thinnest pachymetry (33.92 μm vs. 14.32 μm) in eyes with severe KCN than in eyes with moderate KCN [3]. Gustafsson et al. revealed that eyes with KCN and a K_max_ > 55.0 D had significantly decreased repeatability and greater measurement error than eyes with a K_max_ < 55.0 D [4]. This measurement variability is clinically significant because it can lead to over- or underdiagnosis of keratoconus progression. In particular, measurement variability that makes keratoconic eyes appear less advanced than they are or obscures true progression could lead to delays in recommending sight-preserving treatments such as CXL.

Anterior segment optical coherence tomography (ASOCT) can measure corneal and anterior segment biometry with greater resolution and precision than Scheimpflug tomography. Scheimpflug tomography uses a 1.45-megapixel camera with a rotating 475-nm monochromatic slit of blue light to scan the eye [5]. Swept-source ASOCT can use a 1300-nm tunable near-infrared laser to obtain approximately 50,000 A-scans per second, with deeper tissue penetration, denser sampling acquisition, and higher-resolution imaging [5]. The Anterion ASOCT device’s Cornea App (Heidelberg Engineering, Heidelberg, Germany) obtains 65 radial scans of the cornea, each comprising 256 A-scans per B-scan, providing an axial resolution of <10 μm, a lateral resolution of ≥30 μm, and producing 16,640 corneal topographic measurements of the anterior and posterior surfaces of the cornea [6]. The purpose of this study was to compare the same-session repeatability and inter-device agreement of corneal biometric measurements obtained with Pentacam HR and the Anterion Cornea App in eyes with KCN.

## 2. Materials and Methods

### 2.1. Study Population

From April 2025 through January 2026, patients with a documented diagnosis of keratoconus in at least one eye were identified through electronic health record review before their scheduled clinic visits at the Wilmer Eye Institute clinics in Baltimore and Bethesda, Maryland. We excluded eyes with a documented diagnosis of forme fruste keratoconus, high regular astigmatism without evidence of ectasia, prior keratoplasty, prior corneal cross-linking, or other keratectasias such as pellucid marginal degeneration or keratoglobus. This study was approved by the Johns Hopkins University School of Medicine Institutional Review Board and adhered to the tenets of the Declaration of Helsinki.

### 2.2. Imaging Protocol

For each eye, the same experienced ophthalmic technician acquired three consecutive scans with one device, followed by three consecutive scans with the second device. Device order was neither randomized nor predetermined. All scans were performed in the same room under the same lighting conditions. The patient was directly transferred to the second device after completing the first set of triplicate scans. The intra-device and inter-device imaging intervals were neither standardized nor recorded. Images were acquired within a clinically reasonable interval consistent with the technician’s routine clinical workflow. Technicians were not masked to prior measurements because they were responsible for acquiring and uploading each scan to the Johns Hopkins imaging platform. Since this study was conducted in a high-volume cornea clinic, it was not logistically feasible to routinely reacquire every individual scan displaying an image quality warning. Therefore, no study-specific image quality exclusion criteria were applied. However, when fewer than three complete measurements were available for a given parameter, the eye was excluded from the analysis of that parameter. Measured parameters included anterior and posterior keratometry values (K1, K2, K_mean_, ΔK, and K_max_), central corneal thickness (CCT), and thinnest pachymetry (TP). Corneal biometric variables were abstracted from each imaging report and entered into a REDCap database for data cleanup before statistical analysis [7,8].

### 2.3. Statistical Analysis

KCN severity was characterized using the Belin ABCD grading system and separate K_max_- and TP-based severity classifications. The Belin ABCD system assigns grades from 0 to 4 based on the anterior radius of curvature within a 3.0 mm zone centered on the thinnest corneal point (A), posterior radius of curvature within the same zone (B), thinnest pachymetry (C), and best-corrected distance visual acuity (D) [9]. For parameters A–C, the per-eye grade was defined as the median grade across three consecutive Pentacam HR scans. Parameter D had a single grade per eye derived from best-corrected distance visual acuity. For each eye and device, K_max_ and TP were averaged across three scans and categorized separately using the thresholds reported by Herber et al. [10]. K_max_-based severity was classified as mild (≤50.9 D), moderate (51.0–55.9 D), or advanced (≥56.0 D), and TP-based severity was classified as mild (≥472 μm), moderate (438–471 μm), or advanced (≤437 μm) [10].

Within-device repeatability was assessed separately for each device and parameter. The within-subject standard deviation (Sw) was obtained as the root mean square error of a one-way ANOVA with eye as the grouping factor; the repeatability coefficient (RC = 2.77 × Sw) and coefficient of variation (CV = Sw relative to the grand mean) were derived from it. The intraclass correlation coefficient (ICC) was estimated separately from a three-level mixed-effects model fitted by restricted maximum likelihood (REML), with repeated measurements nested within eyes nested within patients. Within-device repeatability across the keratoconus severity spectrum was displayed using dot plots in which eyes were ranked by their mean value, and each device’s measurement spread was summarized by a smoothed band fitted through the per-eye minimum and maximum readings against the eye.

For inter-device agreement, we compared the mean of three repeated measurements between devices. Inter-device agreement was quantified using Lin’s concordance correlation coefficient (CCC), which jointly captures precision (Pearson r) and accuracy, the bias-correction factor (Cb) [11]. Agreement strength for CCC was interpreted as poor (<0.90), moderate (0.90–0.95), substantial (0.95–0.99), or almost perfect (>0.99), according to McBride (2005) [12]. Z-transform 95% confidence intervals are reported. For visual inspection, we created Bland–Altman graphs by plotting the inter-device difference against the mean of the two device measurements for each parameter. Before deriving limits of agreement (LoA), each parameter was formally tested for proportional bias and heteroscedasticity. When both were nonsignificant, standard fixed LoA, defined as the mean difference ± 1.96 SD, were applied [13]. When either was present, regression-based LoA using the method described by Bland and Altman (1999) was used, allowing the bias and limits to vary across the measurement range [14]. For posterior K1 and posterior K_mean_, the spread intercept was constrained to zero to prevent the limits of agreement from crossing at the extreme end of the measurement range. The 95% confidence intervals for all mean differences and LoA estimates were obtained by clustered percentile bootstrap with 1000 replications and patient-level resampling. The mean inter-device difference was further quantified using a linear mixed-effects model fitted by restricted maximum likelihood (REML), with a patient-level random intercept.

All analyses were performed in Stata version 18.5 SE (StataCorp LLC, College Station, TX, USA). A confidence interval that did not overlap with the null value of 0 and a *p*-value of <0.05 were considered statistically significant.

## 3. Results

### 3.1. Study Population

The study included 28 eyes from 17 patients, with a mean age of 46.00 years. Of patients with available demographic data, 58.82% were male, 41.18% were Caucasian, and 29.41% were African American (Table 1). Mean BCVA was 0.31 logMAR, approximately 20/40 Snellen. Both eyes of one patient were excluded from posterior Ks, CCT, and TP analysis due to missing values from two of the three Anterion acquisitions. Our cohort represented a broad range of KCN severity. According to the Belin ABCD keratoconus grading system (Table 2), parameter A was broadly distributed, with 28.57% of eyes classified as grade 2 and 28.57% as grade 4. Parameter B was weighted toward greater severity, with 50.00% of eyes classified as grade 4. Parameter C was more evenly distributed, with grade 0 being the most common classification (28.57%), whereas grade 3 was the most common classification for parameter D (32.14%). Using the severity criteria reported by Herber et al., the proportion of eyes classified as having advanced disease based on K_max_ was 42.86% with Pentacam HR and 39.29% with Anterion (Table 3). TP-based severity was more evenly distributed across the mild, moderate, and advanced categories with both devices. Overall, the cohort was heterogeneous, with substantial representation of advanced ectatic features.

### 3.2. Measurement Repeatability

As shown in Table 4, both Anterion and Pentacam demonstrated high repeatability, with ICC values > 0.90 and CV values < 5% for all measurements except anterior and posterior ΔK. For anterior ΔK, Anterion and Pentacam had CVs of 15.18% and 19.16%, respectively. For posterior ΔK, Anterion and Pentacam had CVs of 11.71% and 22.03%, respectively. Across all measurements, Anterion demonstrated lower Sw and lower RC compared with Pentacam. For two eyes, posterior keratometry, CCT, and TP values were available from only one of the three repeated scans; these eyes were excluded from the repeatability analysis for these parameters and, consequently, from the agreement analysis as well, because the agreement analysis relied on comparing the mean value across all three measurements between devices.

Anterior K_max_ showed the largest difference in repeatability between the devices. For anterior K_max_ measurements, Anterion had a Sw of 0.50 D and an RC of 1.38 D, compared with a Sw of 0.90 D and an RC of 2.48 D for Pentacam, indicating better repeatability with Anterion. For CCT measurements, Anterion had a Sw of 2.25 μm and an RC of 6.24 μm, whereas Pentacam had a Sw of 6.80 μm and an RC of 18.85 μm. Similarly, for TP measurements, Anterion had a Sw of 2.14 μm and an RC of 5.93 μm, whereas Pentacam had a Sw of 7.34 μm and an RC of 20.34 μm.

For both devices, measurement variability increased as anterior K_max_ increased (Figure 1A) and TP decreased (Figure 1B). However, Anterion consistently demonstrated lower variability than Pentacam across the ranges of K_max_ and TP measurements. A similar pattern was observed for the other pachymetry and keratometry parameters (Supplemental Figure S1A–E, Supplemental Figure S2A–D), with Anterion demonstrating lower variability among repeated measurements compared with Pentacam.

### 3.3. Measurement Agreement

Table 5 shows measurement agreement between the Anterion Cornea App and Pentacam HR. The two devices had substantial agreement for most measurements (CCC > 0.950), except for anterior ΔK (CCC = 0.798) and posterior ΔK (CCC = 0.574), which demonstrated poor agreement. Statistically significant mean inter-device differences were observed for anterior K1, anterior K_mean_, anterior ΔK, CCT, and TP. Compared with Pentacam HR, Anterion measured statistically significantly steeper anterior ΔK values (3.83 vs. 3.15 D, *p* = 0.038), flatter anterior K1 values (46.84 vs. 47.63 D, *p* = 0.003), flatter anterior K_mean_ values (48.65 vs. 49.11 D, *p* = 0.031), thinner CCT values (474.31 vs. 480.40 μm, *p* < 0.001), and thinner TP values (449.46 vs. 461.24 μm, *p* < 0.001). On average, Anterion measured anterior K_max_ 0.71 D flatter than Pentacam HR, although this difference was not statistically significant. Compared with Pentacam HR, Anterion obtained 0.05 D, 0.03 D, and 0.04 D steeper mean measurements for posterior K2, K_mean_, and ΔK, respectively. However, there were no significant differences in mean posterior keratometry measurements between the two devices.

### 3.4. Bland–Altman Analysis

Bland–Altman plots for anterior keratometry are shown in Figure 2. For anterior K2, K_max_, and ΔK, the mean inter-device differences remained close to zero across the range of measured values (Figure 2A–E). After accounting for within-patient correlation, significant proportional bias was present for anterior K1 (*p* < 0.001) and anterior K_mean_ (*p* = 0.018), whereas K2, K_max_, and ΔK showed no significant proportional bias (*p* = 0.965, 0.193, and 0.184, respectively). Significant heteroscedasticity was present for all anterior keratometry parameters (*p* ≤ 0.038), indicating that the scatter of the inter-device differences increased with measurement magnitude. The corresponding limits of agreement, evaluated at the mean measurement, were −2.79 to 1.21 D for K1, −1.81 to 1.62 D for K2, −2.13 to 1.21 D for K_mean_, and −1.61 to 2.98 D for ΔK. For anterior K_max_, inter-device variability increased at values above approximately 54 D, with limits of agreement of −3.97 to 2.55 D, indicating that the two devices’ measurements diverged most in the steepest corneas (Figure 2E).

Bland–Altman plots for posterior keratometry are shown in Supplemental Figure S3. A borderline proportional bias was observed for posterior K1 (*p* = 0.045), whereas no significant proportional bias was observed for posterior K2 (*p* = 0.821), K_mean_ (*p* = 0.121), and ΔK (*p* = 0.057). Significant heteroscedasticity was present for posterior K1 and K_mean_ (both *p* ≤ 0.009), but not for posterior K2 (*p* = 0.180) or ΔK (*p* = 0.176). Limits of agreement were narrow for all posterior keratometry parameters: −0.41 to 0.41 D for K1, −0.55 to 0.46 D for K2, −0.36 to 0.31 D for K_mean_, and −0.60 to 0.67 D for ΔK.

For pachymetry, neither CCT nor TP demonstrated significant proportional bias (*p* = 0.562 and 0.512, respectively) or significant heteroscedasticity (*p* = 0.115 and 0.232, respectively) after accounting for within-patient correlation (Figure 3A,B). These findings indicate that both the magnitude and the scatter of the between-device difference remained constant across the measurement range. The 95% limits of agreement were −23.83 to 11.65 μm for CCT and −40.16 to 16.59 μm for TP.

## 4. Discussion

This study demonstrates that, in eyes with keratoconus, the Anterion Cornea App has greater repeatability and lower same-session measurement variability than Pentacam HR for all corneal tomographic values. This finding is consistent with prior work demonstrating high Anterion repeatability in healthy eyes and extends those observations to keratoconic corneas, in which measurement variability is known to be greater [15]. Although our sample size precluded severity-stratified analysis, the differences in the two devices’ repeatability were more pronounced as K_max_ increased and TP decreased. When comparing the means of three values obtained by the two devices, the devices showed excellent agreement for most tomographic measurements. However, given the differences in same-session measurement variability, the two devices cannot be considered entirely interchangeable. Longitudinal studies incorporating measurement variability across multiple sessions are needed to evaluate how each device performs when monitoring KCN progression.

### 4.1. Keratoconus Assessment

Tomidokoro et al. found that ectatic changes in keratoconus are associated with steepening of both anterior and posterior corneal curvatures, and Yousefi et al. demonstrated that posterior K2 and anterior K_max_ were important keratometric parameters for distinguishing normal eyes from low-grade and high-grade keratoconus [16,17]. In our study, no significant mean inter-device differences were observed for anterior K_max_ and posterior K2, suggesting that the two devices agree on two of the parameters most closely tied to KCN detection. However, other anterior keratometric parameters, including K1, K_mean_, and ΔK, demonstrated systematic inter-device measurement differences. Anterion measured progressively flatter anterior K1 and Kmean values than Pentacam HR as corneal steepness increased. Although these parameters are not typically used alone to assess KCN severity or progression, this proportional bias could confound serial comparisons and supports using the same device across multiple clinic visits. Together with the observed differences in pachymetry and the wider Bland–Altman limits of agreement for several tomographic parameters, these findings indicate that the devices should not be used interchangeably for KCN assessment or longitudinal progression monitoring.

Previous studies have demonstrated that TP is one of the earliest and most sensitive indicators of subclinical keratoconus and that CCT can aid in further stratifying cutoff points in mild KCN [18,19]. Compared with Pentacam HR, Anterion measured TP as 11.99 μm thinner and CCT as 6.16 μm thinner. These inter-device differences are small relative to the broad Amsler–Krumeich pachymetric categories, whose adjacent thresholds are generally separated by approximately 100 μm [20]. However, this mean inter-device offset could become clinically relevant for an eye with a TP measurement within approximately 12 μm of a stage boundary, but this would be present in a minority of cases. Our results also demonstrated that pachymetry measurement variability for both devices increased as corneal thickness decreased. However, this increase was more pronounced with Pentacam HR, whereas Anterion maintained comparatively lower variability in thinner corneas. Anterion’s lower pachymetric variability in thinner corneas suggests that it may reduce uncertainty in distinguishing small changes in corneal thickness from measurement error. Further studies are needed to determine whether Anterion’s increased measurement precision translates into improved or earlier detection of keratoconus.

### 4.2. Inter-Device Agreement and Keratoconus Severity

According to the Amsler–Krumeich classification system, stage III KCN is characterized by astigmatism greater than 8 D, mean central keratometry greater than 53 D, and minimum corneal thickness of 300–400 μm [20]. Multiple studies have also shown that increasing corneal steepness is associated with more advanced keratoconus severity [20,21,22]. Although we were unable to stratify inter-device agreement by keratoconus severity because of the limited sample size, Bland–Altman analysis demonstrated that mean inter-device differences were close to zero in less extreme measurement ranges. However, larger inter-device differences were observed in eyes with steeper keratometry and thinner pachymetry, such as eyes with anterior K_mean_ > 47 D, anterior K_max_ > 54 D, posterior K1 values steeper than −8 D, and TP < 427 μm. These findings support the need to conduct future studies with prespecified severity strata to determine whether disease severity truly modifies inter-device agreement.

### 4.3. Measurement Repeatability

The Global Consensus for monitoring KCN progression recommends tracking changes in anterior curvature, posterior curvature, and corneal thickness [23]. However, a measured change can be interpreted as true progression only if it exceeds the expected variability of the device being used. In our study, Anterion consistently demonstrated lower measurement variability than Pentacam across all keratometric and pachymetric parameters, with the largest differences observed for anterior K_max_, CCT, and TP.

An increase in K_max_ greater than 1.0 D is commonly used as evidence of KCN progression or post-CXL treatment failure [24,25,26]. In our cohort, the RC for anterior K_max_ was 1.38 D with Anterion and 2.48 D with Pentacam. Although both RCs exceed the 1.0 D threshold, the difference between devices is substantial. Pentacam’s RC was approximately 1.0 D higher than Anterion’s, meaning that a 1.5 D increase in K_max_ could potentially represent true progression on Anterion but would fall within expected measurement variability on Pentacam. Anterion’s lower RC may therefore reduce uncertainty when distinguishing true K_max_ increases from device-related variability.

Thinnest pachymetry is incorporated into both the Amsler–Krumeich classification system and the Belin ABCD grading system, and the ABCD progression display uses pachymetric change as one of its parameters for monitoring disease over time [9,20,27]. Prior studies have reported TP repeatability coefficients of approximately 10–20 μm in mild-to-moderate keratoconus and up to approximately 30 μm in severe disease [2,3]. In our study, Anterion demonstrated a TP RC of 5.93 μm, compared with 20.34 μm for Pentacam. Our Anterion repeatability estimates are broadly consistent with those reported by Herber et al., who found SS-OCT to have superior repeatability for pachymetry measurements in eyes with mild and moderate KCN [28]. Herber et al. demonstrated that Anterion’s RC for anterior Ks, posterior Ks, and pachymetric measurements in normal eyes did not exceed 0.4 D, 0.04 D, and 2.15 μm, respectively [28]. Herber et al. also revealed that the RC for anterior Ks, posterior Ks, and pachymetry in normal eyes measured by Pentacam HR did not exceed 0.4 D, 0.1 D, and 4.9 μm, respectively [28]. Compared to our KCN cohort, the repeatability of Anterion and Pentacam HR was greater when measuring normal eyes. Our study extends the findings of Herber et al. by using triplicate rather than duplicate measurements and visualizing measurement variability across a wide range of keratometric and pachymetric values. Anterion’s lower same-session pachymetric variability suggests that smaller true changes in corneal thickness could be distinguished from measurement error. The potential advantages of using Anterion for serial pachymetry measurements to monitor progression in early keratoconus need to be further studied.

### 4.4. Progression Monitoring

Previous studies have reported that measurement variability increases with keratoconus severity, particularly for Pentacam-derived measurements in more advanced disease [2,3,29,30]. Consistent with this, Figure 1 shows that for steeper and thinner corneas, Pentacam HR had greater measurement variability than Anterion, with Anterion maintaining comparatively lower variability across the full range of K_max_ and TP values. Arbelaez et al. similarly demonstrated that measurement repeatability varied by KCN severity, with lower precision in eyes with more advanced disease [30]. Our findings support this observation by demonstrating how the two device platforms’ variability diverges with increasing K_max_ and decreasing TP, which are markers of disease progression.

These differences may be clinically relevant when evaluating candidacy for CXL. CXL is generally performed to stabilize progressive KCN, but it is typically limited to eyes with a minimum corneal thickness of approximately 400 μm to reduce the risk of endothelial toxicity [31]. For patients near this safety threshold, more repeatable pachymetry provides greater confidence that the measured thickness reflects the true value rather than device-related variability. Lower measurement variability may also become relevant after CXL, when clinicians monitor for corneal stabilization or continued progression. Wittig-Silva et al. reported K_max_ flattening of 1.03 D and a 19.52 μm reduction in corneal thickness at 3 years after CXL in eyes with progressive KCN [24]. Changes of this magnitude are well within Pentacam HR’s repeatability coefficients for K_max_ and TP (2.48 D and 20.34 μm, respectively) but are closer to or below those of Anterion (1.38 D and 5.93 μm, respectively). These findings suggest that Anterion may have advantages in detecting subtle post-treatment changes. However, our study was cross-sectional and did not evaluate post-CXL outcomes directly. Longitudinal studies are needed to determine whether Anterion’s improved repeatability translates into better detection of true progression or treatment response.

### 4.5. Strengths and Limitations

This study has several strengths. The prospective, same-visit design ensured that all measurements were obtained on the same day and by the same experienced technician, minimizing inter-operator variability and day-to-day fluctuations as potential confounders. Three consecutive measurements were obtained per device per eye, allowing robust estimation of within-device repeatability using multiple complementary metrics, including ICC, Sw, CV, and RC. The analytic approach addressed both within-device repeatability and between-device agreement, and the use of mixed-effects models accounted for the correlation between fellow eyes within the same patient. The study included eyes across a broad range of keratoconus severity, increasing the clinical relevance of the findings.

This study also has limitations. The sample size was modest, 28 eyes from 17 patients, which limited our ability to perform subgroup analyses by keratoconus severity stage and precluded meaningful comparison of repeatability estimates across our two clinical sites or among individual technicians. Although imaging was performed at two locations with separate devices and multiple technicians, this variability was not formally evaluated as a factor in our analyses because of limited statistical power stemming from the modest sample size. Device order and both intra-device and inter-device intervals were not standardized, which could have introduced order-related or timing-related variability. Previous studies have demonstrated diurnal changes in CCT and TP in eyes with keratoconus over several hours [32]. However, the direct sequential transfer of patients between devices in our study would be expected to reduce the potential effect of such changes. Both eyes from one particular patient’s posterior keratometry, CCT, and TP measurements were only available from one of the triplicate scans. Therefore, this patient’s eyes were excluded from the repeatability and agreement analyses for those parameters. We acknowledge that retaining scans acquired under routine clinical conditions without systematic quality-based reacquisition or exclusion may have increased measurement variability. The limitations in our pragmatic protocol also provide an estimate of the performance of both devices under real-world conditions consistent with routine practice in a high-volume corneal clinic.

Our study assessed same-session repeatability, or test–retest variability within a single visit, but did not assess inter-session reproducibility across multiple clinic visits, which would have captured additional sources of variability relevant to longitudinal KCN monitoring. The cross-sectional design of our study precluded direct evaluation of whether Anterion’s lower measurement variability translates into improved detection of true keratoconus progression or treatment response after CXL. Therefore, the lower same-session variability observed with Anterion should not be interpreted as evidence of superiority for longitudinal progression monitoring. Finally, our comparison was limited to the Anterion Cornea App and Pentacam HR and did not include other anterior segment tomographers, such as the Galilei or Sirius, which may have different repeatability profiles.

## 5. Conclusions

In this study of eyes with KCN, the Anterion Cornea App demonstrated lower same-session measurement variability than Pentacam HR across all keratometric and pachymetric parameters, with the largest differences observed for anterior K_max_ and thinnest pachymetry. We also found decreased inter-device measurement agreement as K_max_ increased and TP decreased. The two devices should not be considered interchangeable for KCN assessment, and clinicians should account for differences in measurement repeatability when interpreting serial measurements for disease progression or eligibility for CXL. Longitudinal studies across multiple sessions are needed to determine whether Anterion’s lower same-session measurement variability improves the detection of true KCN progression.

## Figures and Tables

**Figure 1:**
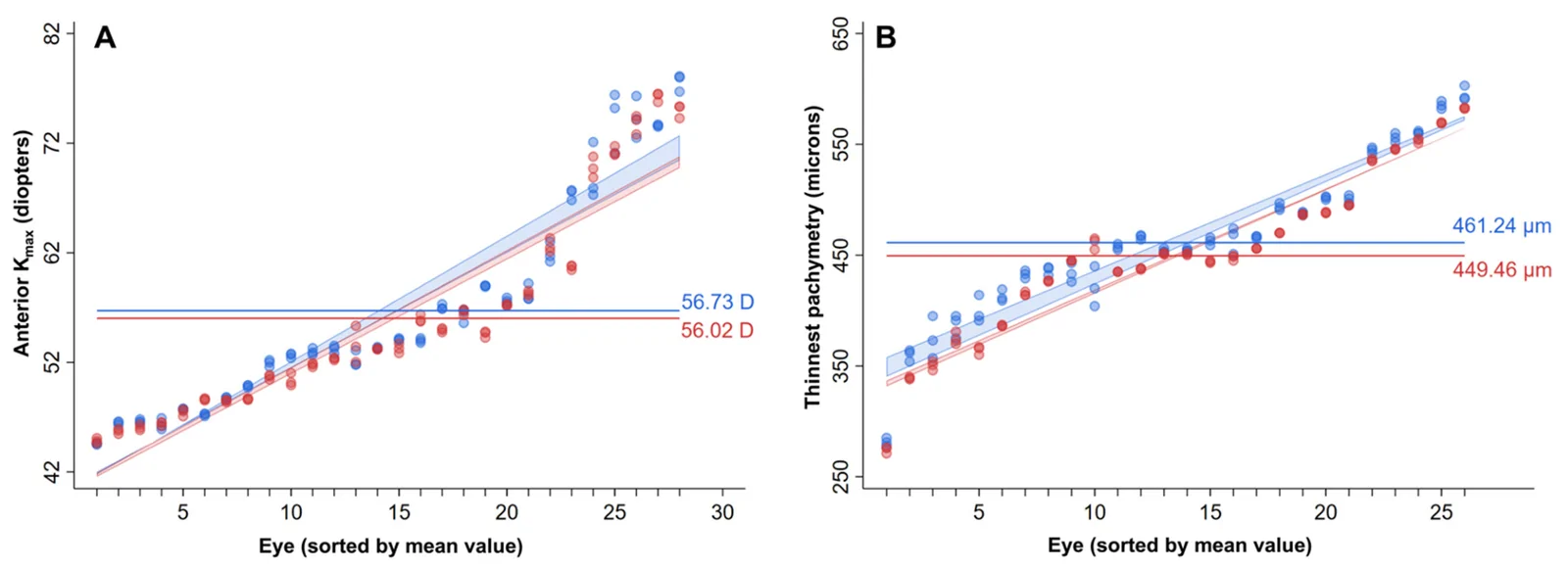
Within-device measurement repeatability across the keratoconus severity spectrum for anterior K_max_ (**A**) and thinnest-point pachymetry (**B**). Eyes are ordered along the x-axis from mildest to most severe based on the mean value across all measurements. Red circles represent individual Anterion scans, and blue circles represent individual Pentacam scans (three scans per eye per device). The shaded bands show the smoothed spread of within-device measurements across the severity spectrum, derived from linear regressions through each eye’s minimum and maximum readings; wider bands indicate greater measurement variability. Horizontal lines indicate the overall device mean, labeled on the right. K_max_: n = 28 eyes; thinnest pachymetry: n = 26 eyes.

**Figure 2:**
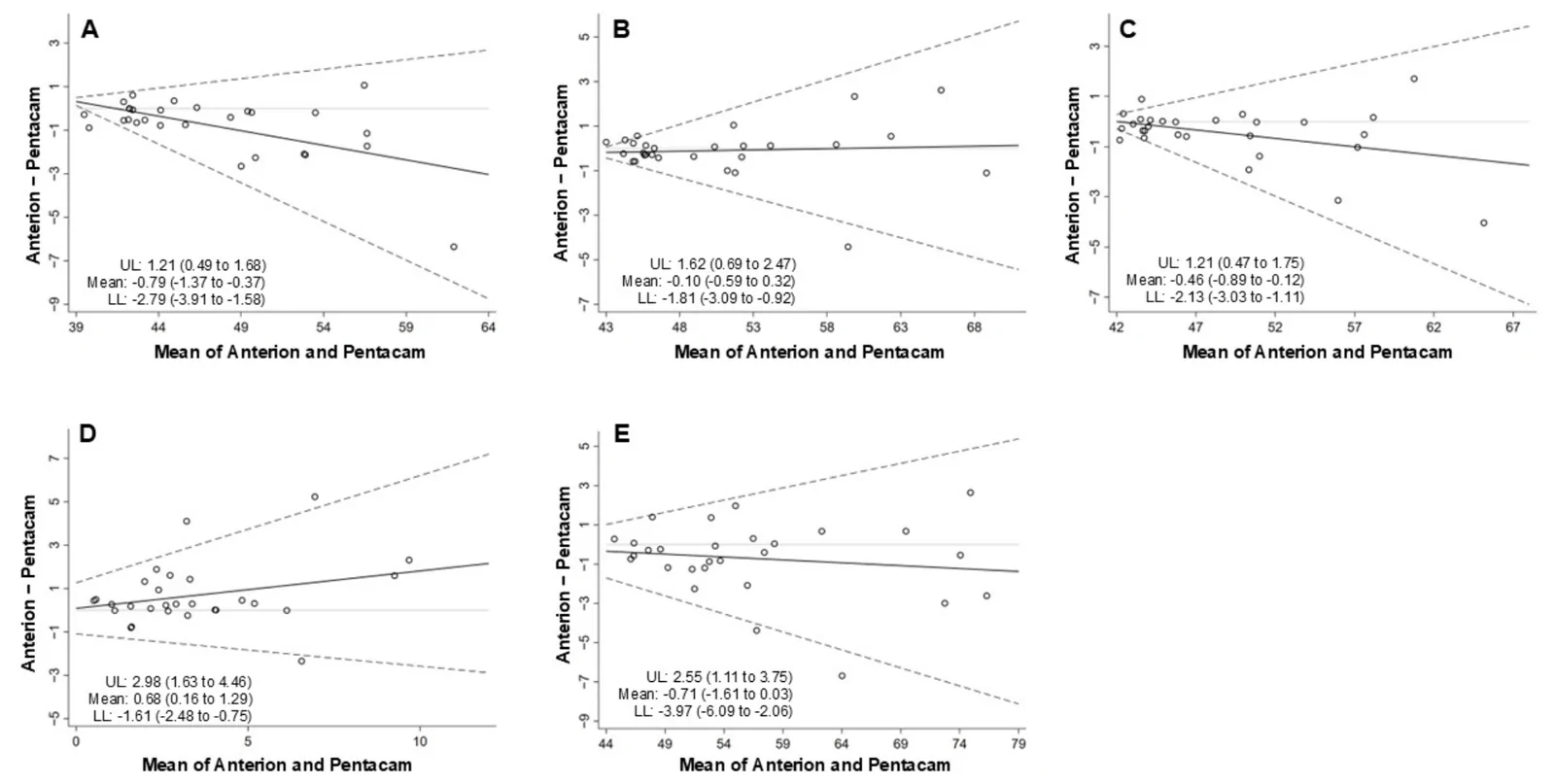
Abbreviations: UL = upper limit of agreement; mean = mean bias; LL = lower limit of agreement. Bland–Altman plots of the inter-device difference (Anterion minus Pentacam) versus the mean of the two devices for anterior K1 (**A**), K2 (**B**), K_mean_ (**C**), ΔK (**D**), and K_max_ (**E**); n = 28 eyes. Hollow circles represent individual eyes. The solid line indicates the mean bias, and the dashed lines indicate the 95% limits of agreement, with the grey line at zero difference. For K1, K2, K_mean_, ΔK, and K_max_, limits were derived using the regression-based two-step method of Bland and Altman, so the bias and limits vary with measurement magnitude [14]. Mean and limits of agreement values inside graphs are reported at the mean of the average measurement. All 95% confidence intervals are stated in parentheses and were obtained by clustered percentile bootstrap (1000 replications, patient-level clustering).

**Figure 3:**
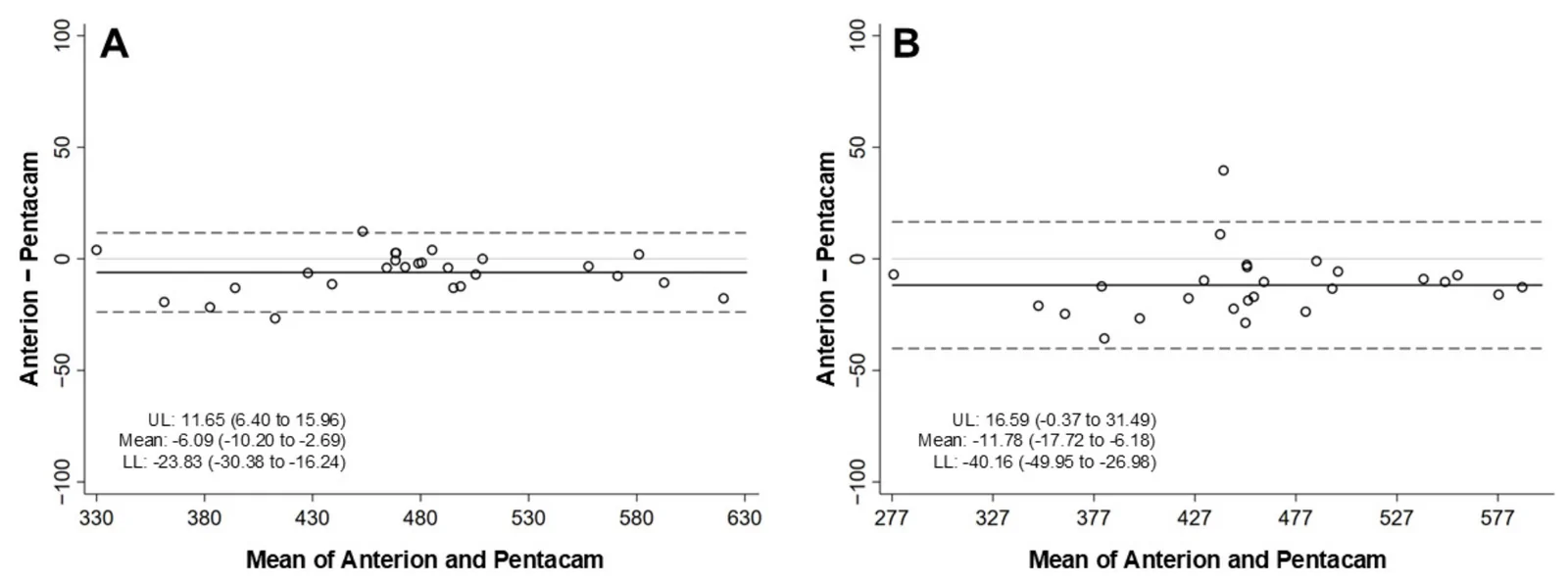
Abbreviations: UL = upper limit of agreement; mean = mean bias; LL = lower limit of agreement. Bland–Altman plots of the inter-device difference (Anterion minus Pentacam) versus the mean of the two devices for central corneal thickness (**A**) and thinnest-point pachymetry (**B**); n = 26 eyes. Plotting conventions are identical to Figure 2. Neither proportional bias nor heteroscedasticity was statistically significant for either parameter, so standard fixed limits of agreement were applied.

**Table 1:** Demographic and clinical characteristics of the study population.

Characteristics	Values
Patients, N	17
Eyes, N	28
Laterality, N (%)	
Left Eye	13 (46.43)
Right Eye	15 (53.57)
Age, Mean (SD)	46.00 (17.68)
Mean BCVA * (SD)	0.31 logMAR (0.29)
Median BCVA * (IQR)	0.18 logMAR (0.10, 0.40)
Gender, N (%)	
Female	7 (41.18)
Male	10 (58.82)
Ethnicity, N (%)	
White	7 (41.18)
Black	5 (29.41)
Asian	1 (5.88)
Unknown	4 (23.53)

Abbreviations: SD = standard deviation; IQR = interquartile range. * Uncorrected VA was used for one eye because the corrected VA was missing.

**Table 2:** Distribution of KCN severity by Belin–Ambrosio ABCD classification.

Grade	A (N = 28)	B (N = 28)	C (N = 28)	D (N = 28)
0	7 (25.00)	3 (10.71)	8 (28.57)	4 (14.29)
1	1 (3.57)	2 (7.14)	4 (14.29)	5 (17.86)
2	8 (28.57)	6 (21.43)	6 (21.43)	6 (21.43)
3	2 (7.14)	1 (3.57)	3 (10.71)	9 (32.14)
4	8 (28.57)	14 (50.00)	5 (17.86)	3 (10.71)
Missing	2 (7.14)	2 (7.14)	2 (7.14)	1 (3.57)

Values are N (%) of eyes with a recorded grade. The Belin ABCD classification system grades each eye 0–4 on four independent parameters [9]. A: anterior radius of curvature in the 3.0 mm zone centered on the thinnest location of the cornea; B: posterior radius of curvature in the 3.0 mm zone centered on the thinnest location of the cornea; C: thinnest pachymetry in μm; D: best-corrected distance visual acuity [9]. The three scan-level grades were identical in 22/26 eyes (84.6%) for parameter A, 23/26 (88.5%) eyes for parameter B, and 23/26 (88.5%) eyes for parameter C; all disagreements spanned one grade except one eye for parameter A (grades 1, 4, and 4). Two eyes of one participant had no grades recorded for parameters A–C, and one further eye had no staging recorded for parameter D.

**Table 3:** KCN severity strata by Kmax and TP measurements from Pentacam HR and Anterion.

Severity	Pentacam HR		Anterion	
	K_max_	TP	K_max_	TP
Mild	8 (28.57)	9 (34.62)	10 (35.71)	8 (30.77)
Moderate	8 (28.57)	7 (26.92)	7 (25.00)	9 (34.62)
Advanced	12 (42.86)	10 (38.46)	11 (39.29)	9 (34.62)

Values are N (%) of 28 eyes for K_max_ and 26 for thinnest pachymetry (TP). Severity was assigned using the cut-offs developed by Herber et al. [10]: Mild: K_max_ ≤ 50.9 D or TP ≥ 472 μm; Moderate: K_max_ 51.0–55.9 D or TP 438–471 μm; Advanced: K_max_ ≥ 56.0 D or TP ≤ 437 μm [10]. K_max_ and TP were averaged across the three scans per eye and per device. Mean values for Pentacam HR: K_max_ 56.73 ± 9.62 D and TP 461.24 ± 72.04 μm; Anterion K_max_ 56.02 ± 9.34 D and TP 449.46 ± 73.97 μm. Two eyes of one participant had only one of three Anterion TP measurements and were excluded from both devices so that the comparison remained paired.

**Table 4:** Repeatability of keratoconus measurements obtained by Anterion and Pentacam HR.

Parameter	Device	N (Eyes)	Mean (SD)	*S_w_*	RC	CV (%)	ICC
Anterior Ks (D)							
K1	Anterion	28	46.84 (5.60)	0.61	1.69	1.31	0.989
K1	Pentacam	28	47.63 (6.39)	0.75	2.09	1.58	0.988
K2	Anterion	28	50.67 (7.19)	0.50	1.40	1.00	0.995
K2	Pentacam	28	50.77 (7.11)	0.61	1.69	1.20	0.994
K_mean_	Anterion	28	48.65 (6.23)	0.49	1.36	1.01	0.994
K_mean_	Pentacam	28	49.11 (6.66)	0.65	1.79	1.32	0.992
ΔK	Anterion	28	3.83 (2.69)	0.58	1.61	15.18	0.955
ΔK	Pentacam	28	3.15 (2.29)	0.60	1.67	19.16	0.936
K_max_	Anterion	28	56.02 (9.34)	0.50	1.38	0.89	0.997
K_max_	Pentacam	28	56.73 (9.62)	0.90	2.48	1.58	0.992
Posterior Ks (D)							
K1	Anterion	26	−6.93 (1.41)	0.07	0.20	1.03	0.998
K1	Pentacam	26	−6.93 (1.32)	0.12	0.36	1.70	0.993
K2	Anterion	26	−7.59 (1.46)	0.11	0.30	1.43	0.995
K2	Pentacam	26	−7.54 (1.46)	0.12	0.33	1.56	0.994
K_mean_	Anterion	26	−7.24 (1.43)	0.08	0.22	1.11	0.997
K_mean_	Pentacam	26	−7.22 (1.38)	0.09	0.26	1.28	0.996
ΔK	Anterion	26	0.67 (0.30)	0.08	0.22	11.71	0.934
ΔK	Pentacam	26	0.63 (0.40)	0.14	0.38	22.03	0.888
Pachymetry (μm)							
CCT	Anterion	26	474.31 (70.71)	2.25	6.24	0.48	0.999
CCT	Pentacam	26	480.40 (69.72)	6.80	18.85	1.42	0.991
TP	Anterion	26	449.46 (73.97)	2.14	5.93	0.48	0.999
TP	Pentacam	26	461.24 (72.04)	7.34	20.34	1.59	0.991

Abbreviations: CCT = central corneal thickness; CV = coefficient of variation; ICC = intraclass correlation coefficient; D = diopter; K = keratometry; RC = repeatability coefficient, which is equivalent to 2.77 × *S_w_*; *S_w_* = within-subject standard deviation; TP = thinnest pachymetry; μm = micrometer.

**Table 5:** Measurement agreement between Pentacam HR and the Anterion Cornea App in eyes with keratoconus.

Parameter	N (Eyes)	Anterion Mean (SD)	Pentacam Mean (SD)	Difference Anterion–Pentacam (95% CI)	*p*-Value	CCC (95% CI)	Cb
Anterior Ks (D)							
K1	28	46.84 (5.60)	47.63 (6.39)	−0.79 (−1.32 to −0.27)	0.003 *	0.964 (0.932 to 0.981)	0.982
K2	28	50.67 (7.19)	50.77 (7.11)	−0.18 (−0.75 to 0.38)	0.527	0.986 (0.970 to 0.993)	1.000
K_mean_	28	48.65 (6.23)	49.11 (6.66)	−0.51 (−0.97 to −0.05)	0.031 *	0.983 (0.965 to 0.991)	0.995
ΔK	28	3.83 (2.69)	3.15 (2.29)	0.71 (0.04 to 1.37)	0.038 *	0.798 (0.627 to 0.896)	0.950
K_max_	28	56.02 (9.34)	56.73 (9.62)	−0.71 (−1.53 to 0.12)	0.093	0.977 (0.951 to 0.989)	0.997
Posterior Ks (D)							
K1	26	−6.93 (1.41)	−6.93 (1.32)	0.001 (−0.09 to 0.10)	0.981	0.984 (0.966 to 0.992)	0.998
K2	26	−7.59 (1.46)	−7.54 (1.46)	−0.05 (−0.18 to 0.08)	0.424	0.984 (0.965 to 0.993)	0.999
K_mean_	26	−7.24 (1.43)	−7.22 (1.38)	−0.03 (−0.12 to 0.06)	0.526	0.990 (0.979 to 0.995)	0.999
ΔK	26	0.67 (0.30)	0.63 (0.40)	0.04 (−0.09 to 0.16)	0.572	0.574 (0.273 to 0.772)	0.952
Pachymetry (μm)							
CCT	26	474.31 (70.71)	480.40 (69.72)	−6.16 (−9.79 to −2.53)	<0.001 *	0.988 (0.974 to 0.994)	0.996
TP	26	449.46 (73.97)	461.24 (72.04)	−11.99 (−17.87 to −6.12)	<0.001 *	0.967 (0.932 to 0.984)	0.986

Abbreviations: CCC = concordance correlation coefficient; Cb = bias correction factor; CCT = central corneal thickness; CI = confidence interval; SD = standard deviation; D = diopter; K = keratometry; TP = thinnest pachymetry; μm = micrometer. Mean difference, CI, and *p*-value for each biometric variable were obtained from the random-effects model. * Statistically significant (*p* < 0.05).

## Data Availability

The data that support the findings of this study are available from the corresponding author upon reasonable request.

## Supplementary Materials

The following supporting information can be downloaded at: https://www.mdpi.com/article/10.3390/bioengineering13101130/s1.
