# Supplementary material for: Measurement Repeatability and Concordance of Corneal Tomography in Keratoconus Using Anterion Versus Pentacam
Source: Bioengineering (Basel). 2026 Sep 28;13(10):1130. doi: 10.3390/bioengineering13101130 (PMC13701922; doi:10.3390/bioengineering13101130)
Supplement: Supplementary file 1 [file bioengineering-13-01130-s001.zip › Supplementary Figure S1.pdf]

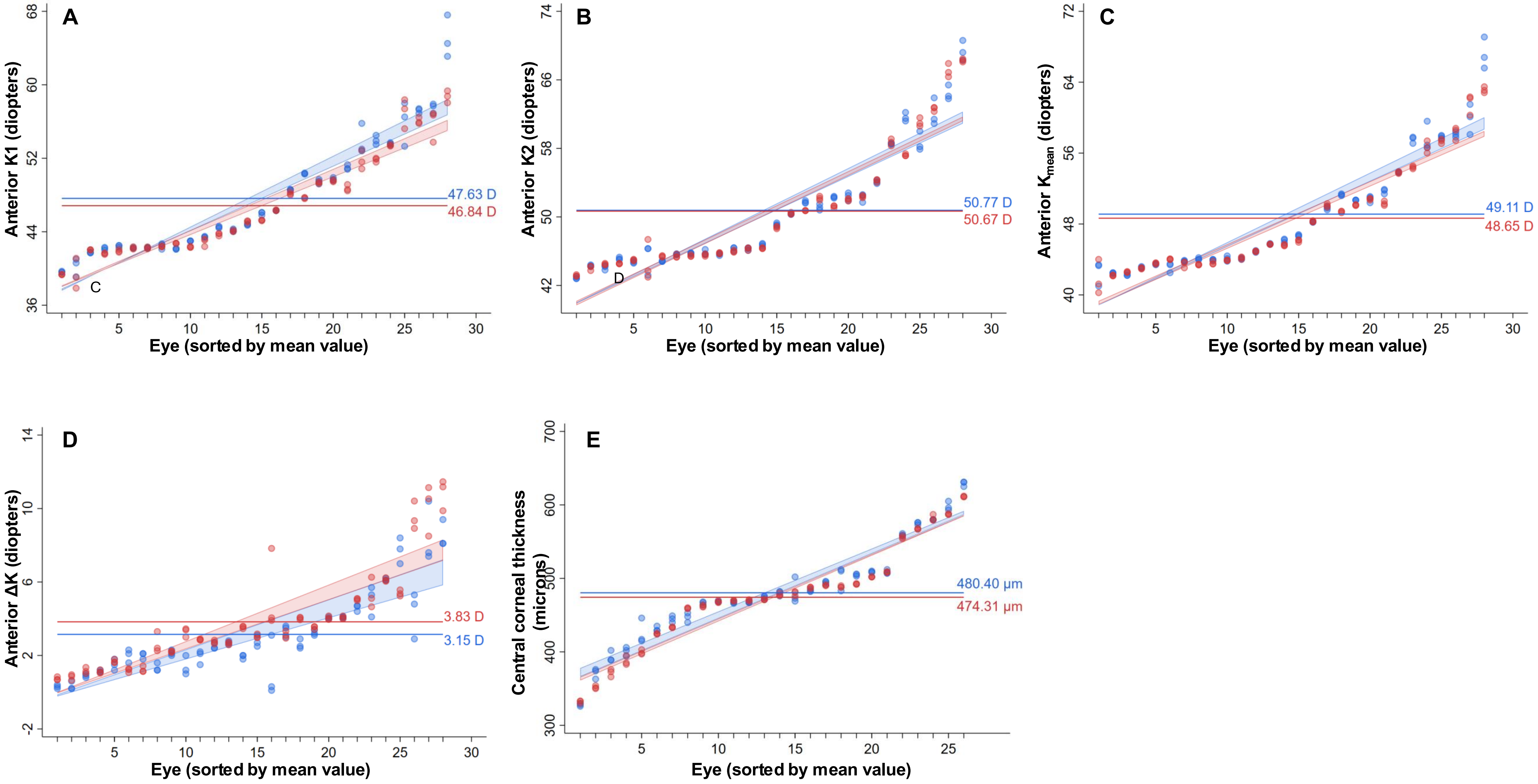
A
Anterior K1 (diopters)
47.63 D
46.84 D
C
Eye (sorted by mean value)
B
Anterior K2 (diopters)
50.77 D
50.67 D
D
Eye (sorted by mean value)
C
Anterior K_{mean} (diopters)
49.11 D
48.65 D
Eye (sorted by mean value)
D
Anterior ΔK (diopters)
3.83 D
3.15 D
Eye (sorted by mean value)
E
Central corneal thickness (microns)
480.40 μm
474.31 μm
Eye (sorted by mean value)
