# Supplementary material for: Measurement Repeatability and Concordance of Corneal Tomography in Keratoconus Using Anterion Versus Pentacam
Source: Bioengineering (Basel). 2026 Sep 28;13(10):1130. doi: 10.3390/bioengineering13101130 (PMC13701922; doi:10.3390/bioengineering13101130)
Supplement: Supplementary file 1 [file bioengineering-13-01130-s001.zip › Supplementary Figure S2.pdf]

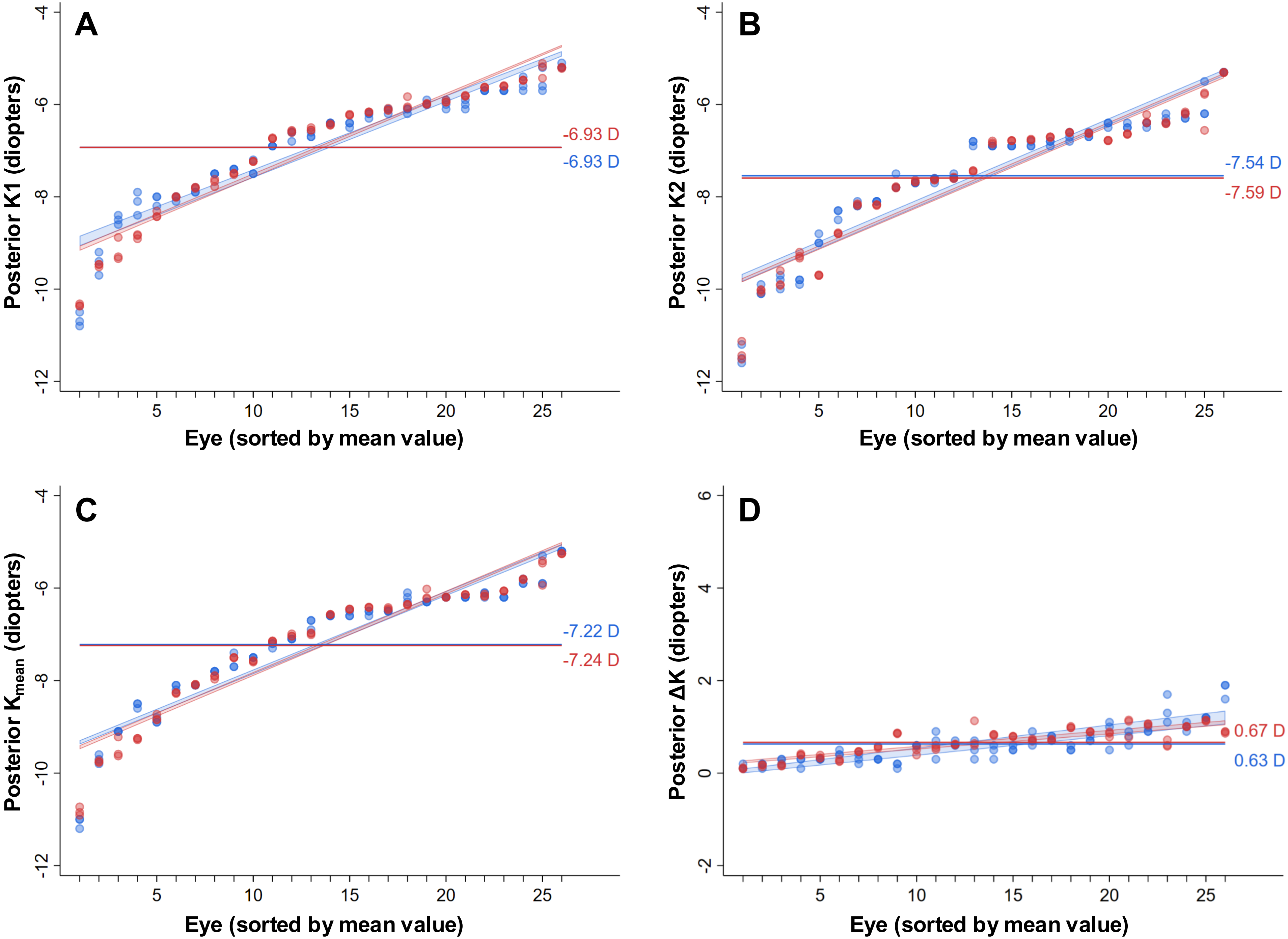
A
Posterior K1 (diopters)
-6.93 D
-6.93 D
Eye (sorted by mean value)
B
Posterior K2 (diopters)
-7.54 D
-7.59 D
Eye (sorted by mean value)
C
Posterior K_{mean} (diopters)
-7.22 D
-7.24 D
Eye (sorted by mean value)
D
Posterior ΔK (diopters)
0.67 D
0.63 D
Eye (sorted by mean value)
