# Supplementary material for: Measurement Repeatability and Concordance of Corneal Tomography in Keratoconus Using Anterion Versus Pentacam
Source: Bioengineering (Basel). 2026 Sep 28;13(10):1130. doi: 10.3390/bioengineering13101130 (PMC13701922; doi:10.3390/bioengineering13101130)
Supplement: Supplementary file 1 [file bioengineering-13-01130-s001.zip › Supplementary Figure S3.pdf]

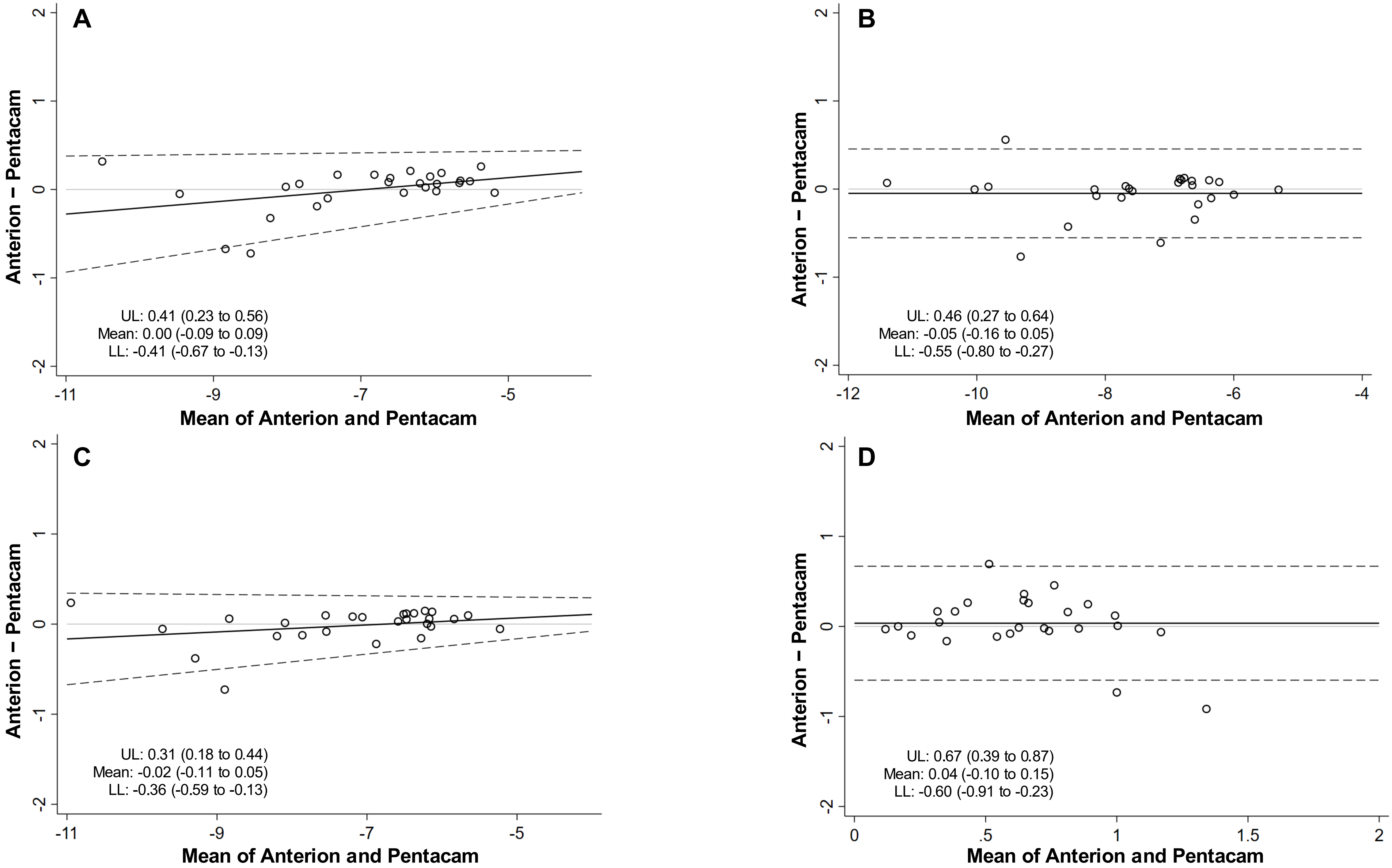
A
UL: 0.41 (0.23 to 0.56)
Mean: 0.00 (-0.09 to 0.09)
LL: -0.41 (-0.67 to -0.13)
Anterion – Pentacam
Mean of Anterion and Pentacam
B
UL: 0.46 (0.27 to 0.64)
Mean: -0.05 (-0.16 to 0.05)
LL: -0.55 (-0.80 to -0.27)
Anterion – Pentacam
Mean of Anterion and Pentacam
C
UL: 0.31 (0.18 to 0.44)
Mean: -0.02 (-0.11 to 0.05)
LL: -0.36 (-0.59 to -0.13)
Anterion – Pentacam
Mean of Anterion and Pentacam
D
UL: 0.67 (0.39 to 0.87)
Mean: 0.04 (-0.10 to 0.15)
LL: -0.60 (-0.91 to -0.23)
Anterion – Pentacam
Mean of Anterion and Pentacam
